# Spatial occurrence records and distributions of tropical Asian butterflies

**DOI:** 10.1038/s41597-025-05333-w

**Published:** 2025-06-13

**Authors:** Eugene Yu Hin Yau, Emily E. Jones, Toby Pak Nok Tsang, Shuang Xing, Richard T. Corlett, Patrick Roehrdanz, David J. Lohman, Adam Kai Chi Lee, Catherine Wai Ching Hai, Shawan Chowdhury, Jane K. Hill, Jade A. T. Badon, Cheong Weei Gan, Yves Basset, I-Ching Chen, Suzan Benedick, Anuj Jain, Tiffany L. T. Ki, Krushnamegh Kunte, Akihiro Nakamura, Lien Van Vu, Sarah A. Scriven, Alice C. Hughes, Timothy C. Bonebrake

**Affiliations:** 1 School of Biological Sciences, The University of Hong Kong, Pokfulam, Hong Kong, China; 2 The University of Toronto Scarborough, 1265 Military Trail, Scarborough, ON M1C 1A4 Canada; 3 School of Ecology, Shenzhen Campus of Sun Yat-sen University, Shenzhen, 518107 China; 4 Center for Integrative Conservation & Yunnan Key Laboratory for the Conservation of Tropical Rainforests and Asian Elephants, Xishuangbanna Tropical Botanical Garden, Chinese Academy of Sciences, Mengla, Yunnan China; 5 Royal Botanic Gardens, Royal Botanic Gardens, Kew, UK; 6 Moore Center for Science and Solutions, Conservation International, Arlington, VA USA; 7 Department of Biology, City College of New York, City University of New York, 160 Convent Avenue New York, New York, NY 10031 USA; 8 PhD Program in Biology, City University of New York, 365 Fifth Avenue, New York, NY 10016 USA; 9 Entomology Section, National Museum of Natural History, Rizal Park, Manila, 1000 Philippines; 10 School of Biological Sciences, Monash University, Clayton, Victoria 3168 Australia; 11 Czech University of Life Sciences Prague, Faculty of Environmental Sciences, Prague, Czech Republic; 12 Biodiversity Society, 49/1 Babar Road, Dhaka, 1207 Bangladesh; 13 Leverhulme Centre for Anthropocene Biodiversity, Department of Biology, University of York, York, YO10 5DD UK; 14 Animal Biology Division, Institute of Biological Sciences, University of the Philippines Los Baños, Laguna, 4031 Philippines; 15 Nature Society Singapore, 510 Geylang Road, Singapore, 389466 Singapore; 16 Smithsonian Tropical Research Institute, Apartado, 0843-03092 Balboa, Ancon Panama; 17 Department of Life Sciences, National Cheng Kung University, Tainan City, Taiwan; 18 Faculty of Sustainable Agriculture, Universiti Malaysia Sabah, Locked Bag No. 3, 90509 Sandakan, Sabah Malaysia; 19 bioSEA Pte Ltd., 68 Chestnut Avenue, Singapore, 679521 Singapore; 20 Science Department, Natural History Museum, London, SW7 5BD UK; 21 Insect Ecology Group, Department of Zoology, University of Cambridge, Cambridge, CB2 3EJ UK; 22 National Centre for Biological Sciences (NCBS), Tata Institute of Fundamental Research (TIFR), GKVK Campus, Bellary Road, Bengaluru, 560065 India; 23 Yunnan Key Laboratory of Forest Ecosystem Stability and Global Change Response, Xishuangbanna Tropical Botanical Garden, Chinese Academy of Sciences, Mengla, Yunnan China; 24 Vietnam National Museum of Nature, Vietnam Academy of Science and Technology, 18 Hoang Quoc Viet, Cau Giay, Ha Noi, Vietnam; 25 School of Biosciences, University of Melbourne, Parkville, Melbourne, Australia

**Keywords:** Ecological modelling, Biogeography

## Abstract

Insect biogeography is poorly documented globally, particularly in the tropics. Recent intensive research in tropical Asia, combined with increasingly available records from citizen science, provides an opportunity to map the distributions of tropical Asian butterflies. We compiled a dataset of 730,190 occurrences of 3,752 tropical Asian butterfly species by aggregating records from GBIF (651,285 records), published literature (27,217), published databases (37,695), and unpublished data (13,993). Here, we present this dataset and single-species distribution maps of 1,576 species. Using these maps, along with records of the 2,176 remaining species, we identified areas of limited sampling (e.g., Myanmar and New Guinea) and predicted areas of high diversity (Peninsular Malaysia and Borneo). This dataset can be leveraged for a range of studies on Asian and tropical butterflies, including 1) species biogeography, 2) sampling prioritization to fill gaps, 3) biodiversity hotspot mapping, and 4) conservation evaluation and planning. We encourage the continued development of this dataset and the associated code as a tool for the conservation of tropical Asian insects.

## Background & Summary

Tropical Asia, home to multiple major global biodiversity hotspots, harbors a rich assemblage of highly range-restricted endemic species^1^. Unfortunately, reliable distribution data for many species in this region are scarce^2^. One prominent challenge for invertebrate conservation, known as the Wallacean shortfall, stems from our inadequate knowledge of species distributions^3^. Insufficient information on species distributions impedes the identification of vulnerable species and the efficient allocation of conservation resources across regions and species^3,4^.

While recent global studies of butterfly biogeography have incorporated data from tropical Asia^5,6^, they have primarily relied on coarse, country-level data to examine biogeographic patterns^5–7^. The distribution information summarized from those data is largely influenced by political boundaries rather than relevant ecological factors and is inadequate for identifying important conservation/vulnerable areas, which requires fine-scale, biogeographic data with low bias^8^. There have also been attempts to map spatial phylogenetic diversity using range maps^9^, but the quality of such spatial analyses is highly dependent on the range maps used, which often fail to capture distribution patterns at local scales, thereby limiting the resolution of the spatial pattern of interest. Although fine-scale geographic distributions of several Asian butterfly groups have been mapped (e.g., *Elymnias* in Wei *et al*.^10^; *Papilio* in Condamine *et al*.^11^; *Polyura* in Toussaint *et al*.^12^; range‐restricted butterflies in Scriven *et al*.^13^), to date, no unified, fine-scale distribution dataset has been produced for the entire region – despite the importance of such a tool for examining patterns of diversity within this highly biodiverse region^1,6^. Existing locality data might not be readily accessible and frequently require aggregation and standardization. Fine-grained information on species distributions is an essential first step for understanding insect biodiversity patterns and conservation needs.

The creation of regional datasets of species distributions is aided by the recent development of large, open-source biodiversity data platforms such as the Global Biodiversity Information Facility (hereafter, GBIF), an online database that organizes crowd-sourced data from citizen science platforms, scientific literature, and specimen collections^14^. These data, however, often include large spatial biases due to uneven sampling and data mobilization efforts among regions^14,15^. Even if available, much of the fine-scale biogeographic data that could be employed to reduce these biases remains buried in literature and regional databases, requiring concerted efforts to make it analysis-ready^7^. Without unified and standardized datasets, it is difficult to test macroecological and macroevolutionary questions^16^, produce high-quality species distribution models^17^, and identify effective conservation targets^5,6,8,18^.

The process of mapping species distributions can be accomplished either through data-driven modeling or by relying on expert knowledge. Expert range maps drawn by experts tend to overestimate occupancy of species at local scales^15,17,19^. In addition, the quality of their source data, hence the uncertainty of the analysis, is often unknown^16^. The dependence of range maps on expert knowledge means this method is available for only a small subset of well-studied species^7^. In contrast, data-driven distribution maps offer greater transparency and reproducibility^18,20^. Modern modeling techniques allow the interpolation of potential distributions into areas for which primary data collection may not be possible, enabling the production of more detailed and reliable distribution maps^3,21^. However, major data gaps exist for occurrence records of most taxa^16,22^, particularly invertebrates, and the non-random distribution of these gaps necessitates careful treatment within models^23^.

Species distribution maps facilitate the identification of species ranges and diversity hotspots. This provides valuable insights for local conservation planning/prioritization^24,25^ and policy-making, paving the way for future investigations into butterfly biogeography^5^ and phylogeographic patterns^24^. Specifically, species distribution maps can guide the allocation of conservation resources, inform the strategic design of protected areas in high-suitability/biologically diverse areas, and identify low-suitability areas in need of management^25,26^, enabling effective conservation interventions. In conjunction with species distribution models (SDMs), occurrence datasets can help inform species reintroduction programs by identifying potentially suitable areas^25,27^ and optimal source populations^28^, and expedite IUCN Red List assessment, which has poor species coverage in Asia. Additionally, applications of SDMs include the modeling of species and community-level responses to climate change^24,27,29^ and the assessment of extinction risks^30^.

**Fig. 1:**
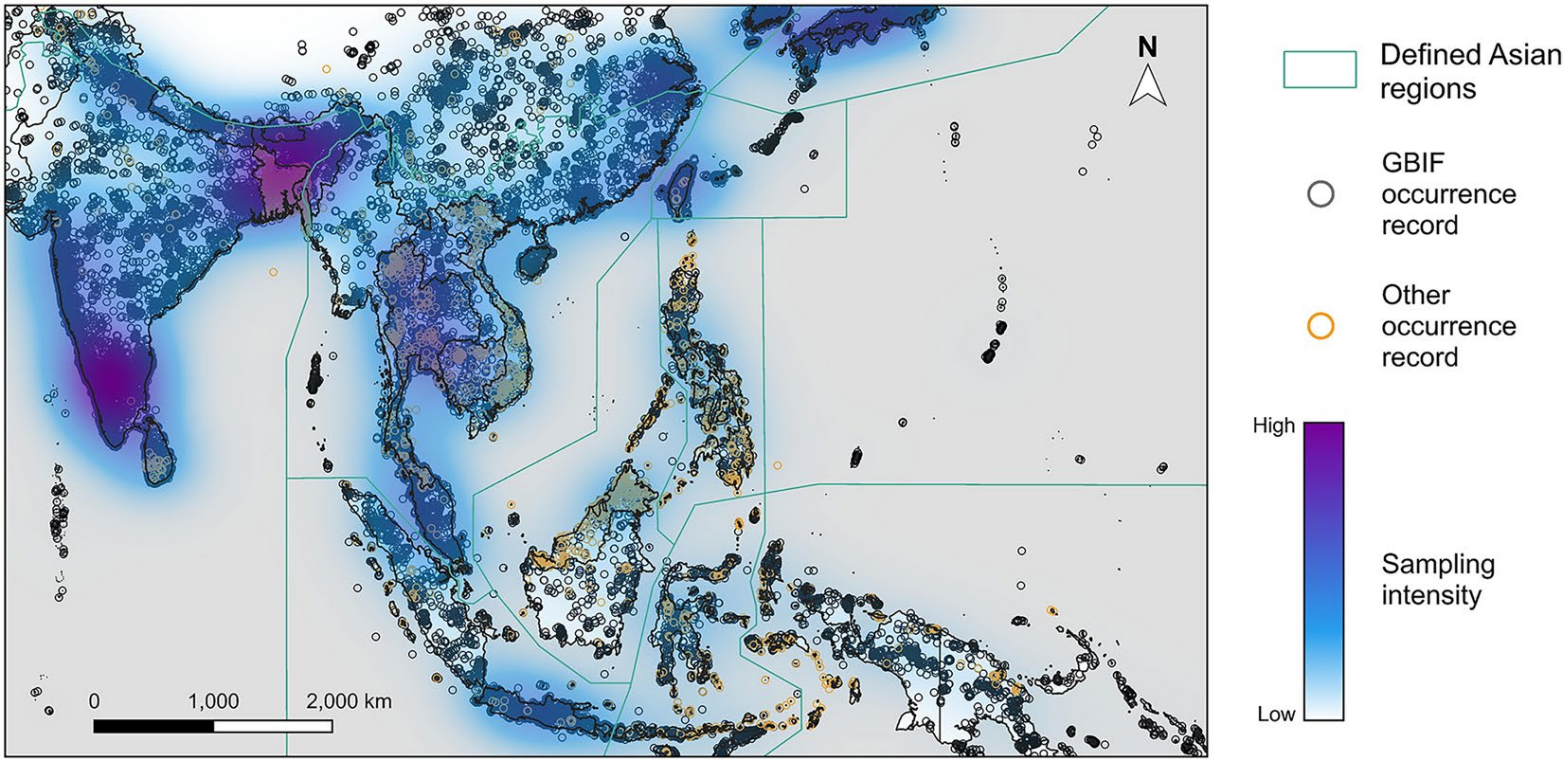
Distribution of GBIF and other occurrence records in our study area. Sampling intensity was estimated by running kernel density on the coordinates of all available occurrence data of every species. Regions of Asian landmasses based on the ecoregions and biogeographic realms as revised by Dinerstein *et al*.^78^, as well as Wallace’s Line, Huxley’s Line, and Weber’s Line.

The need for species conservation is particularly acute in tropical Asia, defined broadly here to include South and Southeast Asia (Fig. 1). The area is home to over 20,000 islands, many of which were repeatedly connected and separated from adjacent landmasses during drastic sea-level fluctuations^31^. This dynamic past led to the evolution of numerous species endemic to single islands or island groups, and as such this region hosts some of the world’s greatest biodiversity – an estimated 15–25% of all well-studied terrestrial taxa and a large proportion of undescribed taxa^32,33^. This highly biodiverse region is also one of the globe’s most biologically threatened: it is estimated that 42% of Southeast Asia’s biodiversity may be lost by 2100 as three-quarters of its primary forests are lost to agriculture, urbanization, and mineral extraction^32,34,35^.

We present a comprehensive dataset of tropical Asian butterfly spatial occurrences, more than half of which are highly accurate (uncertainty < 10 km). This fills a major sampling gap, since Asia is poorly represented in global biodiversity data repositories^15,22,36^; improved datasets are urgently needed to enable effective monitoring and management of biodiversity across the region. Leveraging the data along with tailored SDMs, we generate data-driven distribution maps at a resolution of 10 km × 10 km. These maps enhance a fundamental understanding of butterfly macroecological patterns in tropical Asia. Each butterfly species’ distribution was individually modeled and, together with buffered occurrence points of unmodeled species, employed to assess regional patterns of species diversity. Combined with species distribution models, our aggregated data advances knowledge of butterfly macroecology and facilitates evidence-based decision-making for butterfly conservation in tropical Asia.

## Methods

### Occurrence data

We manually extracted GBIF records on 15 April 2024 for tropical Asian Papilionoidea (Lepidoptera: Hesperiidae, Lycaenidae, Nymphalidae, Papilionidae, Pieridae, Riodinidae; 35.64° N to 11.426° S and 67.588° E to 174.990° E) for the years 1970-present (Derived dataset GBIF.org^37^). The geographical extent of the study area was selected to encompass northern temperate Asia to secure sufficient data to capture the full niche breadth of all species in the subsequent SDMs. We included presence records derived from human observation, preserved specimens, material samples, or literature, provided they had associated coordinates. We omitted all records with >100 km coordinate uncertainty, so-called “fuzzy” taxon matches, and records for which the scientific name was missing or incomplete unless nomenclature could be extracted using a BOLD identifier (boldsystems.org). This resulted in a final number of GBIF records equalling 651,285. The complete metadata, filtering methods, and data usage information for this GBIF-derived dataset is available GBIF^37^ (10.15468/dl.9wyfb6).

Roughly 73% (472,714) of these records are ‘research-grade’ observations from iNaturalist. Information on how this designation is made is available at GBIF.org. The accuracy of opportunistically collected data from crowd-sourced platforms like GBIF is often diminished due to misidentifications, taxonomic, spatial, and temporal biases, as well as uneven taxonomic validation due to lack of standardized reference data^14,38–41^. Given these potential issues, and to fill geographic gaps, we supplemented these GBIF data with expert data (coauthor datasets, published literature) and harmonized binomials to a single expert dataset (Lamas, 2015. Catalogue of the butterflies (Papilionoidea), available from the author.; see below).

We extracted data from the B2D2 Database of Butterflies for Borneo provided by JKH/the Darwin Initiative (n = 19,417) (https://www-users.york.ac.uk/~jkh6/index.htm), a dataset for Bangladesh provided by SC (Chowdhury *et al*.^42^; n = 18,278), and unpublished datasets from coauthors AN, DJL, LVV, TK, and YB (n = 13,993). For geographic regions with relatively few records (e.g., China, Myanmar, Thailand) and for species with < 10 records, we conducted targeted searches of post-1970 published literature on Google Scholar in English and Chinese (simplified and traditional) (genus OR genus + species + country name), producing an additional 27,217 records. Although some publications lacked collection dates for records (e.g., checklists), we assume that the inclusion of species in recent publications is indicative of species’ current localities. Data sources for all records are provided in the reference column (C) in Occurrence Records of Tropical Asian Butterflies: 1970–2024.csv and alphabetically in Data Sources for Occurrence Records of Tropical Asian Butterflies: 1970–2024.pdf at our Figshare repository^43^.

For all records in published sources, we extracted coordinates, locality name, locality type (e.g., exact coordinates, city, national park, island, or province), country, and year of record (where available). If exact coordinates were not provided by the source, we used Google Earth Pro (v7.3.6.9345) to estimate the locality centroid for any record provided at the province level or below (e.g., national park or city). For records from islands ≤ 100 km at the widest dimension (e.g., localities within the Philippines and Indonesia), we estimated the island or archipelago centroid. If a range of coordinates was provided (e.g., records from *The Butterflies of Vietnam*), we selected a point within the range.

Final binomial harmonization, validation, and authority assignment were conducted by DJL using a taxonomic reference prepared by Gerardo Lamas (Lamas, 2015). Family names were aligned to GBIF.

The resulting database^43^ (Occurrence Records of Tropical Asian Butterflies 1970–2024) consists of 730,190 occurrence records for 3,752 species from 551 genera. These records represent approximately 19.2% of all described butterfly species globally^44,45^ (around 19,500 spp. according to Lamas (2015); but see Pinkert *et al*.^5^). Records of Nymphalidae (1,357 spp.; 316,584 records) comprise 43% of the dataset, followed by Lycaenidae (1,046 spp; 145,521 records), Papilionidae (270 spp.; 102,309 records), Pieridae (409 spp.; 97,722 records), Hesperiidae (636 spp.; 61,107 records), and Riodinidae (34 spp.; 6,947 records). Of the 3,752 species in the database, 1,631 (43.5%) are represented by ≥ 10 records within the extent of 36° N to 10° S and 69° E to 161.6° E that are > 10 km apart (see details on distribution modeling below).

Most occurrence records are concentrated in a limited number of regions, for example, India (26.97% of all data), Taiwan (13.08% of all data), Singapore (8.48% of all data), Hong Kong (7.89% of all data), and Malaysia (6.82% of all data) (Fig. 1). Equatorial regions together with southern China are relatively underrepresented in our dataset. As much of the data is derived from GBIF, which contains a large proportion of citizen science data, we observed a clustering of our data in areas of high human population density and a general lack of data in more inaccessible regions.

### SDM methods and results

Five algorithms, Generalized Linear Model (GLM), Maximum Entropy (MaxEnt), Multivariate Adaptive Regression Splines (MARS), Classification Tree Analysis (CTA), and eXtreme Gradient Boosting (XGBOOST) were selected to create an ensemble model for each butterfly species, using the ensemble platform “biomod2”^46^ in R. We ensured that the underlying mechanism of our selection of algorithms was diverse and relatively balanced between the main categories of algorithms. We used 13 predictor variables for selection by individual models. All modeling was conducted at 10 km × 10 km resolution.

The Generalized Linear Model (GLM) is a regression-based algorithm widely used in SDMs^47^. They are not as flexible when fitting complex response curve shapes, but this also means that GLMs are less vulnerable to overfitting^47^. Maximum Entropy (MaxEnt) in our study was based on the “maxnet” R package^48^, which uses penalized maximum likelihood for model fitting. MaxEnt is one of the computationally less expensive algorithms that perform well, making it a popular SDM algorithm^49^. MaxEnt is more capable of fitting complicated, non-linear response curves, enabling users to model more complex relationships by using progressively complex statistics based on the number of samples available^50^. The classification tree analysis (CTA) used by our SDM is based on the “rpart” R package^51^. The CTA algorithm recursively splits one group of data into two subgroups using one of the predictor variables given; therefore, the final model can be visualized as binary decision trees^51^. Finally, eXtreme Gradient Boosting (XGBoost) is one of the more computationally efficient gradient boosting algorithms implemented in R by the “xgboost” package^52^. Boosting algorithms feature an ensemble of weak models, each trained to minimize the errors of the previous models^47,53^.

**Fig. 2:**
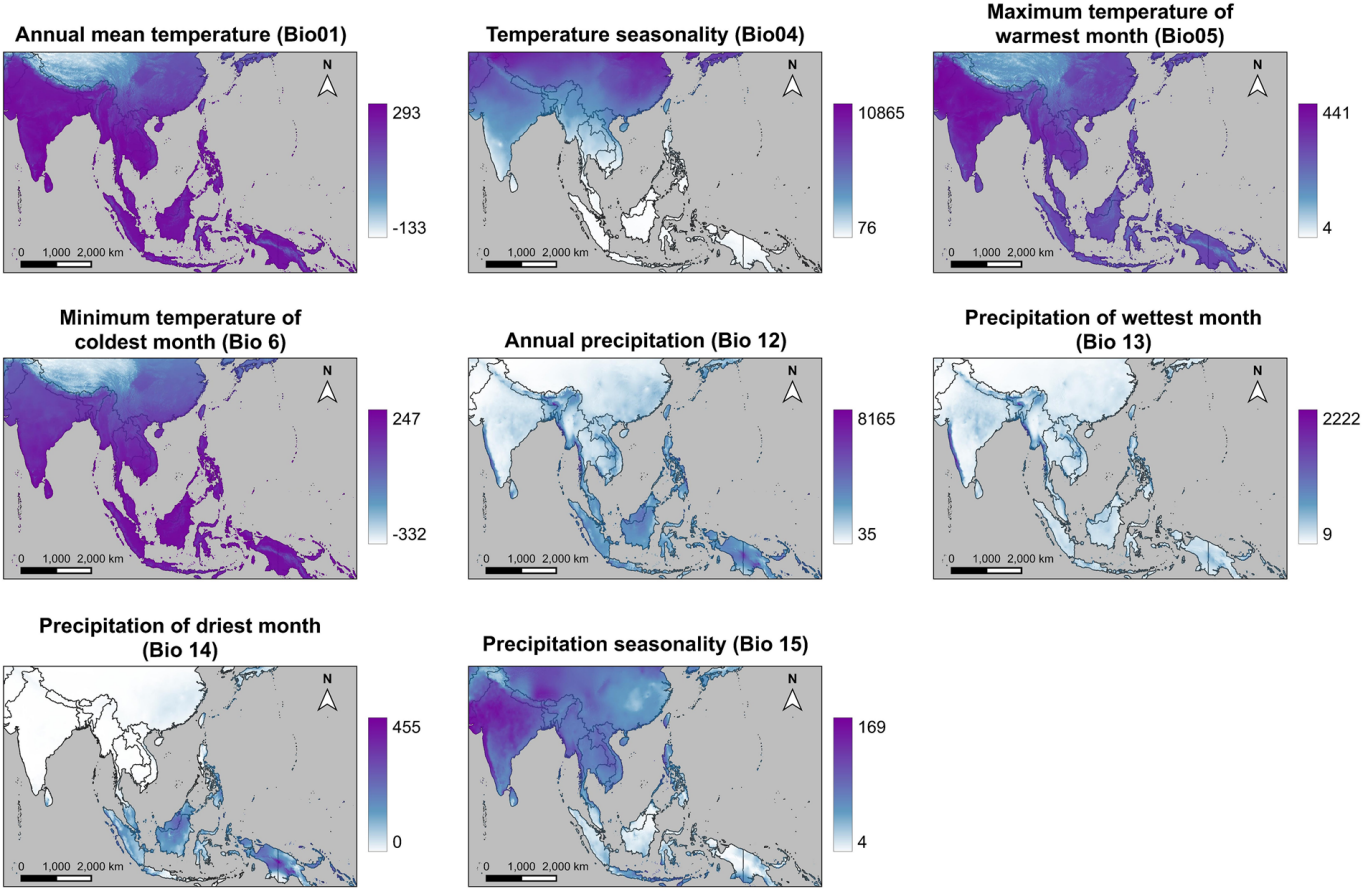
Climatic predictor variables included in our SDMs.

**Fig. 3:**
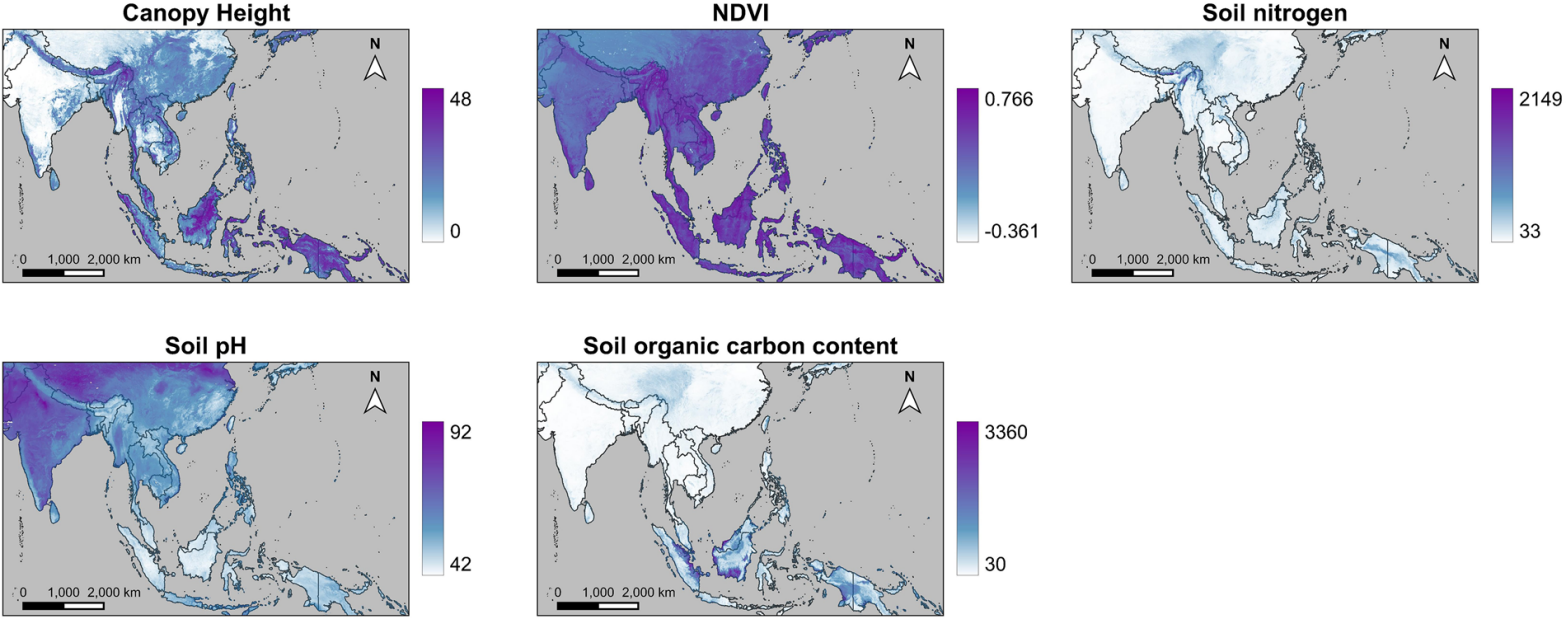
Non-climatic predictor variables included in our SDMs.

For the species distribution models, we used 13 predictor variables, which comprised 8 Bioclim variables extracted from WorldClim^54^ (see Fig. 2), three soil variables extracted from SoilGrids^55^ through ISRIC (International Soil Reference and Information Centre)^56^ (see Fig. 3), and 2 vegetation variables derived from satellite data (see Fig. 3). The Bioclim variables employed included annual mean temperature (Bio 1), temperature seasonality (Bio 4), maximum temperature of warmest month (Bio 5), minimum temperature of coldest month (Bio 6), annual precipitation (Bio 12), precipitation of wettest month (Bio 13), precipitation of driest month (Bio 14), precipitation seasonality (Bio 15). The soil variables at a depth of 5–15 cm were used, including soil pH (phh2o), soil organic carbon content in the fine earth fraction (SOC), and total nitrogen (nitrogen). Nitrogen is generally recognized as one of the main limiting elements for plant growth^57^, while soil organic carbon indicates soil quality^58^. In addition, soil pH exerts considerable influence on soil biogeochemical processes, ultimately impacting plant growth^59^. The selection of variables for our models was guided by expert knowledge to reflect/cover the key limitations and resources relevant to both butterflies and their host plants. Knowledge of the study region and biology/ecophysiology of the species being modeled allows the identification of the most ecologically relevant variables; therefore, it is the preferred approach for variable selection^47,49,60,61^.

The vegetation variables used were the Normalized Difference Vegetation Index (NDVI) and Canopy Height. NDVI was calculated from the USGS Landsat 5 (Level 2, Collection 2, Tier 1, 1985 – 1999) and USGS Landsat 7 (Level 2, Collection 2, Tier 1, 2000 – 2020) datasets, with a customized script to filter satellite images by cloud cover (retaining images with 15% or less cloud cover over land) and to obtain the mean NDVI value. Canopy Height data was retrieved from the ETH Global Sentinel-2 10 m Canopy Height dataset^62^. These vegetation cover variables were directly used to model the land cover/habitat available to butterflies. Mean NDVI provided information on the general greenness of an area, while Canopy Height data offer structural details on vegetation to better identify different types of habitats. Together, these variables indicate resource availability and, to some extent, habitat structure. To address potential issues associated with negative values in NDVI data, an alternative variable, Corrected NDVI, which contains no negative values, was also examined. The Corrected NDVI is derived from the equation Corrected NDVI = NDVI + 1. However, the SDMs using Corrected NDVI produced identical results to those using standard NDVI data, indicating that our models were unaffected by negative NDVI values.

The resolution of all environmental variables was set to 10 km × 10 km by averaging the values from contributing grid cells. This resolution was chosen as a result of balancing the spatial accuracy of available data and computational capabilities. Our data includes 441,356 records with coordinate uncertainty data, while an additional 288,834 records do not have coordinate uncertainty data. Among the records with known coordinate uncertainty, 80,374 (18.21% of records with uncertainty data) had uncertainties ranging from 1–10 km, and 39,302 (8.90% of records with uncertainty data) had uncertainties exceeding 10 km, thus, 10 km seemed a reasonable compromise to reflect this. For the construction of SDMs, the map of the study area and predicting variables were formatted to share the same extent, resolution, and projection. Next, the map of tropical Asia and all explanatory variable rasters were all projected to equal area projection EPSG:6933 and cropped to the extent of 36° N to 10° S and 69° E to 161.6° E to fully cover the study region. The final, cleaned dataset used in our SDMs included 721,335 global records.

We used a function to further prepare the input files required by biomod2 and to generate SDMs individually for each species. Occurrence data of a species was first extracted from our butterfly occurrence dataset and used to produce a raster of resolution of 10 km × 10 km. A total of *n* cells in the raster were assigned a value of “1” to represent at least one occurrence record present in that cell, while cells with no record were assigned “n/a” instead of “0” since no true absence data is available.

Only species with n ≥ 10 were modeled. It has been shown that SDMs based on ten occurrence points can reach 90% of the maximum possible accuracy^63^, while recent studies suggest a minimum requirement of 3 to 13 occurrence points in virtual simulations and 14 to 25 occurrence points in real-world conditions to infer accurate SDMs^64^. Therefore, *n = 10* was chosen as the lower limit of sample size for constructing SDMs to maximize the number of species modeled while maintaining a reasonably high predictive accuracy^63^. A total of 1,631 species met this qualification, whereas 1,951 species had fewer records. For each species, occurrence records were split into three sets: 10% of the data was first reserved for model evaluation, and another 10% was then partitioned for model validation, leaving the remaining 80% of data for model calibration. The partitioning of model validation data was repeated 5 times to generate five different combinations of calibration and validation occurrence data.

Before SDM construction, pseudo-absence records were generated. Despite our efforts to fill the spatial data gaps, the sampling effort of our dataset is still spatially biased toward highly populated areas and roads due to the overwhelming number of records from GBIF and iNaturalist in our dataset (more than 80%). As part of our effort to account for biases in our data, we integrated the spatial bias of our dataset into the generation of pseudo-absence records, assuming that all species were sampled in areas with at least one occurrence record of any species. To capture such spatial bias, we created a raster layer of the spatial sampling effort for all species across our study area (shown as sampling intensity in Fig. 1), which is equivalent to the bias layer commonly used in the MaxEnt program. This was done by pooling occurrence data of all species used in our models and summarising them in a raster, then performing two-dimensional kernel density estimation (kde2d) using the R package “MASS”^65^ with the default settings. We excluded cells with occurrence records and sampled the remaining study area for pseudo-absence records based on the bias layer, giving more weight to well-sampled areas, as suggested by Phillips *et al*.^66^ and Ferrier *et al*.^67^. Following the recommendation of Barbet‐Massin *et al*.^68^, for calibration, validation, and evaluation data, we produced five sets of pseudo-absence data for each species, maintaining a 1:1 ratio between the number of pseudo-absence points and occurrence points in each set.

Subsequently, we constructed SDMs for each species using five different partitions of calibration and validation occurrence data, five selected algorithms, and five sets of pseudo-absence data. This resulted in a total of 125 SDM models (5 × 5 × 5). Both presence and pseudo-absence records were given equal weight during model construction to ensure a consistent prevalence of 0.5 among all species. We applied a generalized setting for all butterfly species for consistency across species, with adjustments made only to the learning rate and the number of decision trees for the XGBoost algorithm to address overfitting. Other model tuning options were retained at their default.

We generated binary outputs by maximizing True Skill Statistics (TSS), a widely used threshold-dependent index of model fit. Ensemble modeling was selected over single best models for its superior performance in rare species^69^, and its robustness to uncertainties in individual models by capturing the central tendency among models^47,70,71^. We constructed an ensemble model using all single models with TSS values greater than 0.7, ensuring that only “substantial” models were included^72^. A total of 1,576 species out of the 1,631 modeled species obtained one or more single models meeting such criteria, allowing the further construction of ensemble models. The ensemble model was generated using the mean algorithm^71^, where all candidate models’ probabilistic predictions were averaged without weighting. Finally, we projected the ensemble model to the current environment using the same variables when constructing the SDMs.

Ensemble models were evaluated using two metrics: TSS and Boyce index. TSS and Kappa are two of the most popular SDM threshold-dependent evaluation metrics. TSS was chosen over Kappa due to the inherent dependency of Kappa on species prevalence^73^. Since we are modeling thousands of species with differing degrees of rarity and prevalence, TSS is more appropriate for model comparison between species. TSS varies from +1 to −1, in which +1 indicates perfect agreement with evaluation data, while a TSS value close to or less than 0 indicates model performance comparable to a random model^73^.

Following the suggestions of Hernandez *et al*.^74^ and Breiner *et al*.^69^ to use multiple evaluation measures when using presence-only data, we also calculated the Boyce index for all models built to supplement TSS. The Boyce index is capable of providing an accurate and reliable measure of model performance for models based on presence-only data^75^, which is the key reason for its use in our study. Another reason for the use of the Boyce index is its lower sensitivity (correlation) to species prevalence relative to other metrics, including CVI, MaxKappa, and adjusted D2^75^, while AUC and TSS also have a negative correlation with prevalence^73^. AUC was also found to produce inflated estimates of model quality when the modeled species is rare^76^. Boyce index ranges from +1 to −1, in which +1 indicates the model is of the highest quality and perfectly predicts evaluation data, while −1 indicates counter-prediction of evaluation data^75^. Boyce index with a value close to 0 indicates the model performs no better than a random model^75^.

**Fig. 4:**
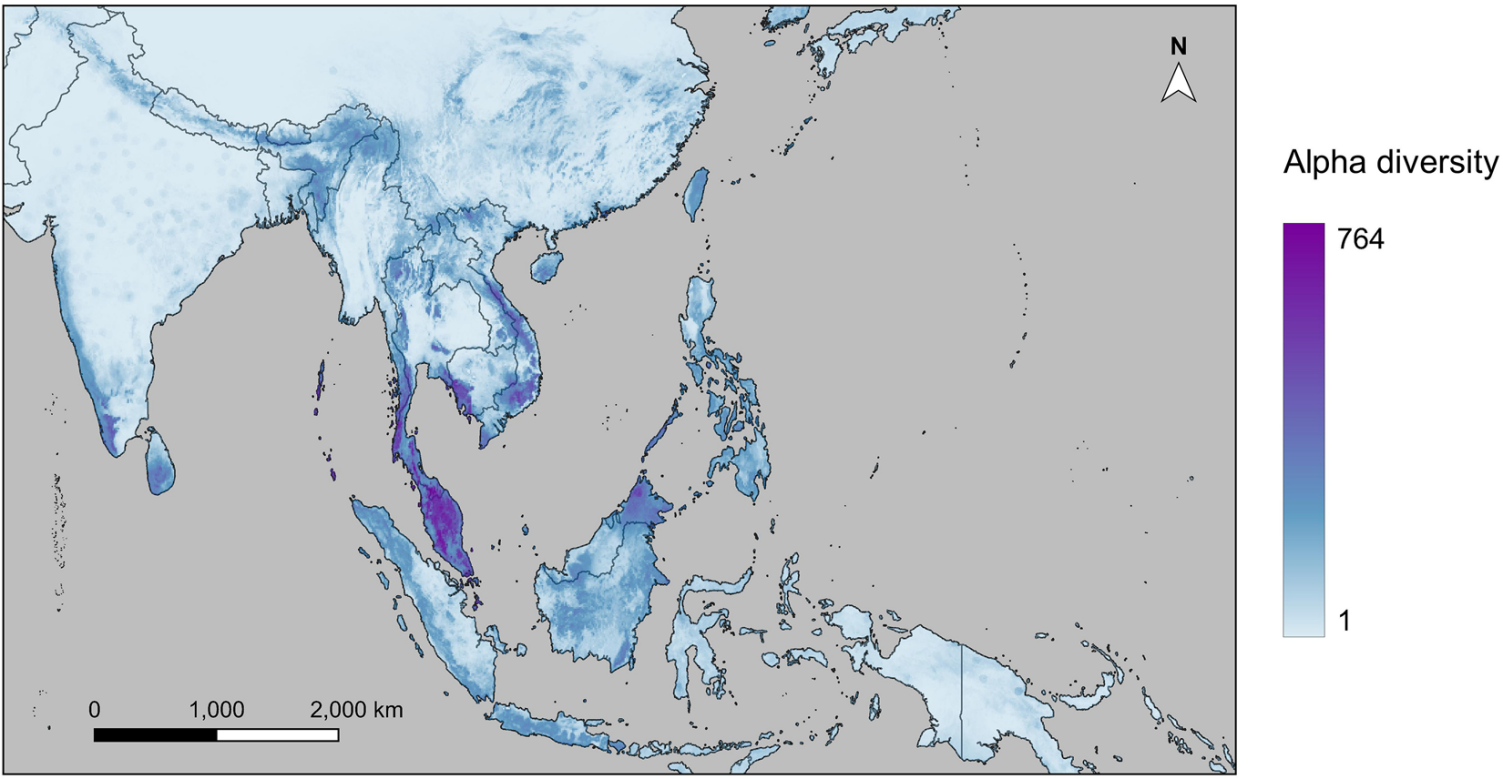
Projected distribution of butterfly diversity based on our species distribution models, using the mean algorithm for ensemble modeling.

To factor biogeography into predictions and correct for biogeographic overprediction generated by our SDMs (and account for differences between fundamental and realised niches), we restrained the sampling of pseudo-absence records and distribution maps produced by our models to regions that hosted more than 1% of species points (as such regions fall within species biogeographic ranges) following the methods of Zhou *et al*.^77^. By incorporating biogeography into model predictions, we aimed to reflect the impact of oceans as dispersal barriers in the SDM outputs to give a more realistic estimate of species’ distribution and reduce false positive predictions. We first divided the landmasses of tropical Asia into 11 biogeographic regions (Fig. 1) based on the ecoregions and biogeographic realms as revised by Dinerstein *et al*.^78^, as well as Wallace’s Line, Huxley’s Line, and Weber’s Line. For each species, we identified regions that included at least 1% of the species occurrence records, considering them to be “active regions”. We then cropped the SDM-predicted distribution maps to include only the active regions specific to each species. These cropped distribution maps were stacked together to generate an alpha diversity map, which illustrates the number of species present in each 10 km × 10 km cell across tropical Asia. The stacked SDM predictions highlighted a number of locations with relatively high diversity, exceeding 600 species in some locations (Fig. 4).

### Point buffer methods

A total of 2,176 species (58.0% of all recorded species in our dataset) were excluded from our species distribution modeling outputs either due to insufficient data or low species distribution model quality. Out of the 2,176 species without valid SDM outputs, we plotted and buffered occurrence records of 2,070 (55.2% of all recorded species in our dataset) species which have at least 1 valid occurrence point within the range of 36° N to 10° S and 69° E to 161.6° E to infer alpha diversity. We first mapped their occurrence records and created 30 km-wide polygons (buffers) around these points to enhance clarity. Subsequently, the buffered occurrence points were converted into binary raster maps for each species and stacked to generate an additional alpha diversity map, representing species with limited occurrence records.

**Fig. 5:**
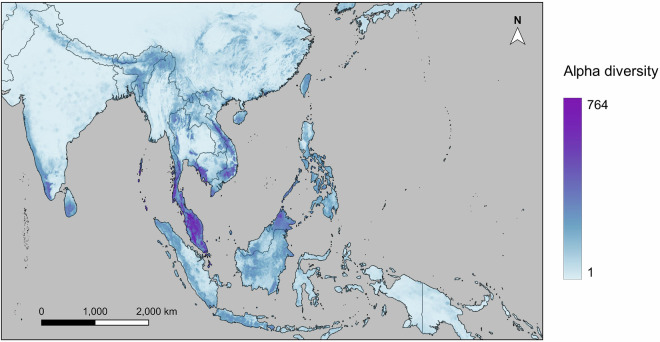
Estimated distribution of butterfly diversity based on our species distribution model projections and buffered occurrence points (for species not included in our SDM outputs).

The diversity map derived from buffered occurrence points was then stacked with the species distribution model (SDM) projections to produce Fig. 5. This figure provides an overview of the alpha diversity of all species documented in our dataset. We identified two major butterfly diversity hotspots: peninsular Malaysia and the Sabah region of Borneo. We also found high levels of diversity predicted in Borneo, Sumatra, coastal Cambodia, southern Thailand, the Western Ghats in peninsular India, the Assam region of India, the Cardamom Mountains in Cambodia, and Vietnam.

### Minimum convex polygon (MCP) methods

Among the 2,176 species without valid SDM outputs, there were 46 species with occurrence data widely distributed within the biogeographic regions they inhabit. Distributional constraints of such widespread species can challenge effective models^76^, as reflected by their low SDM validation scores (all constructed SDMs failing our TSS > 0.7 requirement), which eventually resulted in the exclusion of their SDM outputs. To address this issue, we also computed minimum convex polygon (MCP) for each of the 46 widespread species. MCPs were first computed using all available occurrence records of the species concerned (after excluding those dated before 1970). To factor in dispersal barriers (biogeography), the estimated distributions (MCPs) were limited to biogeographic regions (Fig. 1) with at least ten occurrence records of the relevant species. The MCP outputs are accessible on the Figshare repository^43^ for reference but were not used to generate any of the diversity maps in this paper (these species are instead accounted for via the point buffers).

### Software

We calculated the SDMs in R^79^, version 4.1.2. To construct and merge the SDMs into ensemble models, we utilized the “biomod2” package, version 4.2-4^46^. The high-performance computing cluster HPC2021 at The University of Hong Kong, operating on CentOS 8, was employed to run the SDMs.

## Data Records

**Fig. 6:**
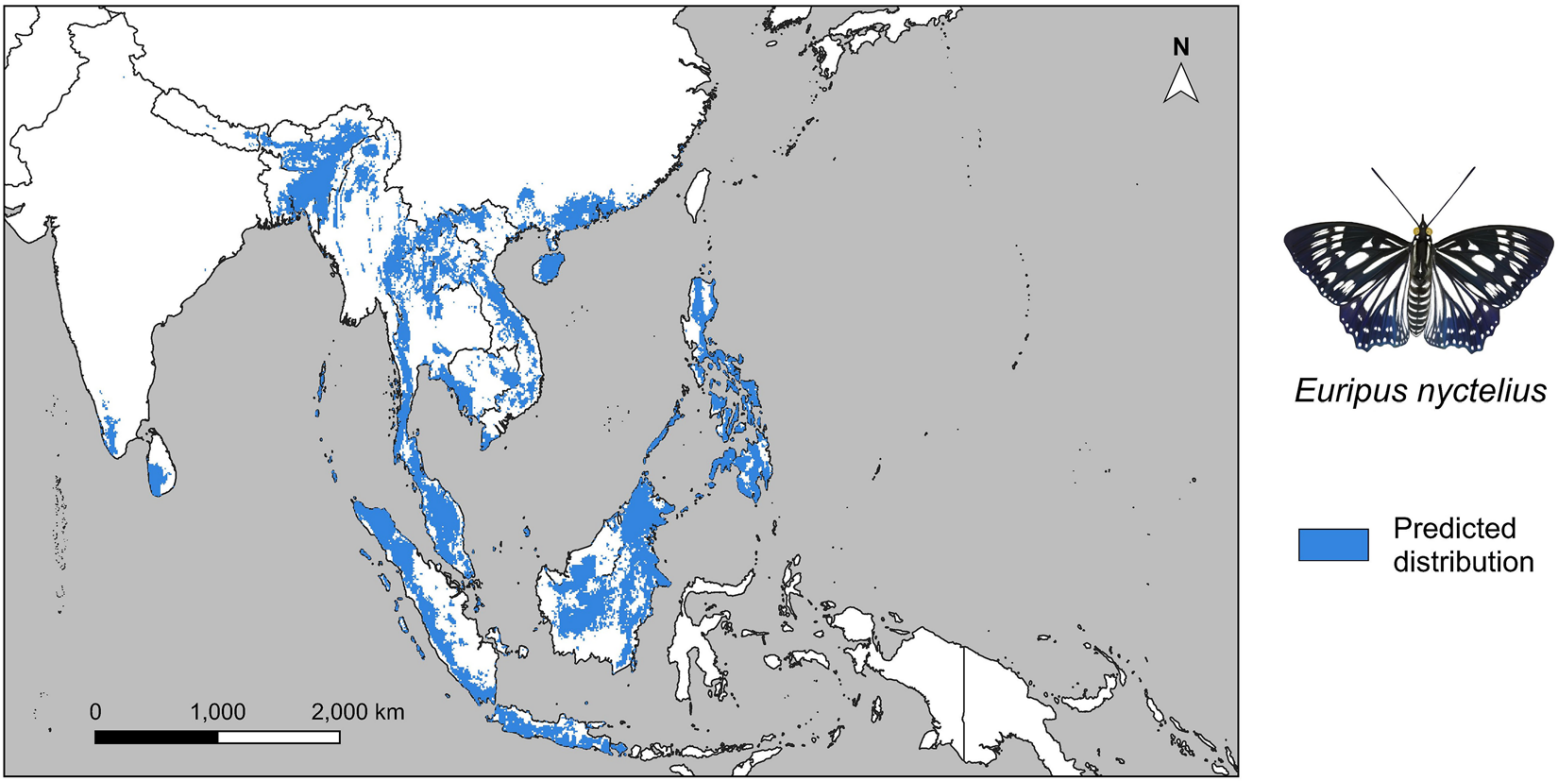
As a case study for the single species maps, an SDM-predicted distribution of *Euripus nyctelius* (Doubleday, 1845) (Nymphalidae: Apaturinae) based on our occurrence dataset is displayed.

All data, including Occurrence Records of Tropical Asian Butterflies 1970–2024 (.csv), Metadata for Occurrence Records of Tropical Asian Butterflies 1970–2024 (.xlsx), Data Sources for Occurrence Records of Tropical Asian Butterflies 1970–2024 (PDF), SDM-predicted single species distribution maps (as individual.tif files, e.g., Fig. 6, or as one single PDF file), single species buffered occurrences (as individual.tif files or as one single PDF file), single species minimum convex polygons (as individual.tif files or as one single PDF file), and documentation on the basis (SDM projection/MCP/Occurrence record buffer) of range maps for individual species (sp_output_type.csv) are available from our Figshare repository^43^ (10.6084/m9.figshare.25037645). These outputs are licensed under a CC BY 4.0 license. The GBIF-derived dataset (downloadable as TSV file under a CC BY-NC 4.0 license), associated metadata, contributing datasets, and information about our data filtering methods are available at GBIF^37^ (10.15468/dl.9wyfb6).

## Technical Validation

### SDM model evaluation/verification

The mean TSS score of all ensemble models is 0.899, with a standard deviation of 0.222, while the Boyce index is 0.729, with a standard deviation of 0.325. Both evaluation metrics indicate that the models constructed are of good quality. The mean TSS score of our ensemble models is higher than 0.8, falling into the category of “almost perfect” models according to the widely used division suggested by Landis & Koch^72^ (e.g., Capinha *et al*.^80^; Jones *et al*.^81^). Since we only included models with TSS values of more than 0.7 in our ensemble models, a high mean TSS score among the ensemble models is expected. The mean Boyce index of our models is higher than 0.7, which has been considered an indicator of good models in other studies (e.g., Rupprecht *et al*.^82^). Boyce index value of 0.5 is usually considered a cutoff for acceptable performance^83^.

### Collaborator evaluation

Our model outputs were also inspected by experts to evaluate their plausibility. Plausibility checks form an important part of model validation by making sure the modeling results confine to the known range and possible range of the species modeled^49,84^, serving as a supplement to evaluation metrics, which only measure the goodness of fit of models.

Experts (coauthors/collaborators) agreed that our model outputs are generally reasonable and informative. However, it is important to note that some of the sampling biases persisted in the final model outputs despite our efforts to address data gaps by incorporating additional datasets. We, therefore, encourage future data contributions to improve the coverage of our dataset, especially in the areas with identified data gaps.

Although the majority of data gaps can be attributed to insufficient sampling effort, the relative absence of data in the Philippines (and potentially other parts of tropical Asia) is primarily a result of the dominance of Facebook over other platforms like iNaturalist for citizen science data contribution. However, such data on Facebook contains limited information since EXIF data (containing GPS coordinates) of photos are removed when uploaded. Mining occurrence data with valid location records from Facebook (e.g., Chowdhury *et al*.^18^) and other sources may also provide useful data.

Our modeling results identified the Cardamom Mountains on the Cambodian-Thai border as a butterfly diversity hotspot. During the Pleistocene when sea levels were up to 120 m lower than present, and this area was on the eastern edge of a paleoriver watershed that included the similarly diverse Malay peninsula and extended south to present-day Borneo^85,86^. The high diversity in this area is likely relictual^87^. Endemism in this area likely contributes to high butterfly diversity, which supports our models’ prediction there.

Multiple experts pointed out the unexpected diversity differences between different parts of Borneo. While our models identified Sabah as a hotspot for butterfly diversity, lower diversity was predicted for other parts of Borneo, such as Sarawak and Kalimantan. This contradicted our expectations, as all these areas possess mountainous regions and endemic species, suggesting similar levels of butterfly diversity. The heart of Borneo, characterized by lower disturbance compared to other parts of the island, was also predicted to host a relatively lower diversity of butterflies by our models. Such a model prediction also contradicts our expectation of higher butterfly diversity in less disturbed areas. This inconsistency between expected and modeled butterfly diversity in Borneo may be attributed to sampling bias, evident through the alignment of modeled butterfly diversity with political boundaries and sampling intensity (Fig. 1), and the lower modeled diversity in less accessible areas such as the heart of Borneo (Figs. 4, 5). The lack of data in less accessible areas has been discussed by Hughes *et al*.^15^ and Boakes *et al*.^88^, while this trend is even more obvious in citizen science data^15^, which constitutes a considerable proportion of our dataset. However, the peak in butterfly diversity observed in northern Borneo does reflect the higher botanical richness in that area as modelled by Raes *et al*.^89^.

While some of the spatial variations in the sampling effort of our dataset are reflected in the spatial bias of our modeling results, there are several notable discrepancies between the distribution of data and modeled diversity. Figure 1 illustrates that Japan, Taiwan, and northern Thailand have a relatively high intensity of sampling effort compared to their predicted butterfly diversity in Fig. 4. Conversely, a reversed pattern is evident in Southern Borneo and Southern Sumatra, where our data shows low sampling effort but our models predict high butterfly diversity. These patterns demonstrate the robustness of the models to some of the spatial sampling biases present in our data.

To determine the variable importance in our SDMs, we calculated the mean variable importance for each variable throughout the ensemble models of all species. Temperature seasonality (Bio 4) emerged as the most important variable (scoring 0.235 out of 1), followed by the minimum temperature of the coldest month (Bio 6, scoring 0.138 out of 1), annual mean temperature (Bio 1, scoring 0.111 out of 1) and Canopy Height (scoring 0.109 out of 1). Precipitation of driest month (Bio 14, scoring 0.0955 out of 1), Soil pH (phh2o, scoring 0.0927 out of 1), and precipitation seasonality (Bio 15, scoring 0.0918 out of 1) also exhibited high importance in the models. The ranking of variable importance in the SDMs conforms to the hierarchical framework of Pearson & Dawson^90^, in which climatic variables exert greater control over species distribution at continental scales, while land cover and soil variables gain influence at more local scales. In addition, the high importance of temperature variables, particularly temperature seasonality (Bio 4), is consistent with the results of Carvalho *et al*.^91^, which highlighted the strong impact of temperature, especially temperature seasonality, on butterfly distribution and diversity.

### Cross-validation with published literature

**Fig. 7:**
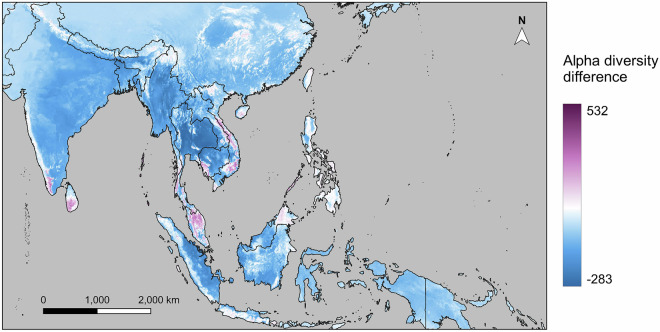
Differences between our SDM results and that of Daru (2024)^92^ based on modelled alpha diversity of butterflies in tropical Asia. Our SDMs predicted presence of more species than that of Daru (2024)^92^ in areas shown in purple (positive values in diversity difference), while SDMs constructed by Daru (2024)^92^ predicted presence of more species than ours in areas shown in blue (negative values in diversity difference).

We compared the alpha diversity raster predictions made by our SDMs (α_1_) with that of SDMs recently published by Daru (2024) (α_2_), which map global butterfly species’ distributions (Fig. 7). A total of 1,354 butterfly species modeled by both studies were identified. Using the modeled distributions of these shared species, an alpha diversity map was generated for each study. Differences between SDM outputs was calculated by the equation α_1_ - α_2_ for every raster cell.

Most of the areas where our SDMs predict lower alpha diversity than that of Daru (2024)^92^ correspond to areas with low Canopy Height and NDVI values (Fig. 3), except for Sulawesi and New Guinea. Areas where our SDMs predict higher alpha diversity generally have relatively high Canopy Height or NDVI values, most of them contributing to diversity hotspots identified by us. Differences between our diversity results vs. Daru (2024)^92^ are caused by different underlying datasets as well as distinct modeling decisions for the SDMs.

## Usage Notes

The predictor variables considered in our SDMs, which include the eight Bioclim variables and the three SoilGrids variables, are products of interpolation between available point data^54,55^. As with most data collected without stratified sampling, these point data are likely to be spatially biased towards densely populated and developed regions for the Bioclim variables^54^, and agricultrual areas for the SoilGrids variables^55^. Users should note that our SDMs inherit some of these biases, as well as uncertainties in the interpolation result. In particular, the range of butterflies dependent on narrow-ranged host plants might be underestimated.

By generating more pseudo-absences for SDMs in well-sampled areas with the use of the bias mask, we are essentially augmenting the weighting of extensively surveyed regions in our models, while unsampled habitats may be presumed as suitable. Consequently, the transferability of our models to unsampled areas is limited, especially when extrapolating in novel environments not covered by training data^66^ or in areas where biogeographic barriers prevent dispersal. This is also one of the reasons for restraining our model predictions to the regions where a species is known to occur so that the results are not overly optimistic. Such an approach to pseudo-absence generation also assumes that the data collection method is consistent throughout the entire dataset^66^, while our dataset is compiled from various sources. To use our data and models for the prediction of future butterfly distribution under climate change, we suggest using the “random” method from the biomod2 package to generate pseudo-absence records.

Regarding uncertainty in model results, we have limited confidence in the model predictions for some regions, e.g. New Guinea and Sulawesi (Fig. 1) due to a lack of samples and the unique biogeography of the islands. The presence of biogeographic barriers such as Wallace’s Line and Huxley’s Line further restrict the use of occurrence data from other regions to infer butterfly distribution in these specific areas. In addition to model uncertainties, the biogeographic barriers incorporated in our single species distribution outputs will, in reality, have variable impacts on species (given variation in dispersal ability for different species). Therefore, we suggest that users of the single species distribution maps (based on either SDM projections or MCPs) exercise caution and interpret the outputs with awareness of these limitations.

The butterfly occurrence and projected distribution data holds the potential for a wide range of further analyses. For example, overlaps between areas of high butterfly alpha diversity and Protected Areas (PAs) and Key Biodiversity Areas (KBAs) could shed light on gaps in conservation effort. Endemicity should also be further investigated. While we identified butterfly alpha diversity hotspots, areas and regions with relatively lower butterfly alpha diversity should not be overlooked in conservation planning, especially those hosting highly endemic butterfly species such as Sulawesi (239 endemic butterfly species, 42.9% of total species^93^) and Papua New Guinea^94^ (e.g., more than 60% of butterfly taxa in New Britain were reported to be “regionally endemic”^95^).

## Data Availability

All code used to conduct synonym harmonization, preprocess environmental variables for SDMs, execute SDMs, process SDM outputs, and conduct point buffer analysis can be accessed in our GitHub project repository: https://github.com/eugeneyau/Tropical-Asian-Butterfly-Distribution.
